# Supplementary material for: Pan-vaccine analysis reveals innate immune endotypes predictive of antibody responses to vaccination
Source: Nat Immunol. 2022 Oct 31;23(12):1777–87. doi: 10.1038/s41590-022-01329-5 (PMC9747610; doi:10.1038/s41590-022-01329-5)
Supplement: Supplementary file 1 — Reporting Summary [file 41590_2022_1329_MOESM1_ESM.pdf]

nature portfolio

Corresponding author(s): Rafick-Pierre Sekaly

Last updated by author(s): August 30, 2022

Reporting Summary

Nature Portfolio wishes to improve the reproducibility of the work that we publish. This form provides structure for consistency and transparency in reporting. For further information on Nature Portfolio policies, see our Editorial Policies and the Editorial Policy Checklist.

Statistics

For all statistical analyses, confirm that the following items are present in the figure legend, table legend, main text, or Methods section.

n/a	Confirmed	
☐	☒	The exact sample size (*n*) for each experimental group/condition, given as a discrete number and unit of measurement
☐	☒	A statement on whether measurements were taken from distinct samples or whether the same sample was measured repeatedly
☐	☒	The statistical test(s) used AND whether they are one- or two-sided<br>*Only common tests should be described solely by name; describe more complex techniques in the Methods section.*
☐	☒	A description of all covariates tested
☐	☒	A description of any assumptions or corrections, such as tests of normality and adjustment for multiple comparisons
☐	☒	A full description of the statistical parameters including central tendency (e.g. means) or other basic estimates (e.g. regression coefficient) AND variation (e.g. standard deviation) or associated estimates of uncertainty (e.g. confidence intervals)
☐	☒	For null hypothesis testing, the test statistic (e.g. *F*, *t*, *r*) with confidence intervals, effect sizes, degrees of freedom and *P* value noted<br>*Give P values as exact values whenever suitable.*
☒	☐	For Bayesian analysis, information on the choice of priors and Markov chain Monte Carlo settings
☐	☒	For hierarchical and complex designs, identification of the appropriate level for tests and full reporting of outcomes
☒	☐	Estimates of effect sizes (e.g. Cohen's *d*, Pearson's *r*), indicating how they were calculated

Our web collection on statistics for biologists contains articles on many of the points above.

Software and code

Policy information about availability of computer code

Data collection	ImmPort (software release June 2022) and ImmuneSpace (software release December 2021) were used to collect the transcriptomic and phenotypic data used in the manuscript.
Data analysis	R (version 4.2.0) and Bioconductor (version 3.14) were used to generate the figures of the manuscript. MSigDB (version 7.2) was used for pathway analysis. MetaIntegrator (version 2.1.3) was used to test previous pre-vaccination signatures. UMAP of the umap python package (version 0.1.1) was used for dimension reduction of the scRNASeq data.

For manuscripts utilizing custom algorithms or software that are central to the research but not yet described in published literature, software must be made available to editors and reviewers. We strongly encourage code deposition in a community repository (e.g. GitHub). See the Nature Portfolio guidelines for submitting code & software for further information.

Data

Policy information about availability of data

All manuscripts must include a data availability statement. This statement should provide the following information, where applicable:

- Accession codes, unique identifiers, or web links for publicly available datasets
- A description of any restrictions on data availability
- For clinical datasets or third party data, please ensure that the statement adheres to our policy

The data used in the paper can be found at www.immunespace.org/is2.url

Human research participants

Policy information about studies involving human research participants and Sex and Gender in Research.

Reporting on sex and gender	Participants of all sexes were used in this study. For participants with missing sex, mRNA expression of genes on the Y chromosome was used to infer sex.
Population characteristics	Healthy participants agesd 18 years and above were included.
Recruitment	Not applicable. A meta-analysis of the publicly available data was performed in this manuscript.
Ethics oversight	Not applicable. No recruitment or clinical trial were performed in this manuscript.

Note that full information on the approval of the study protocol must also be provided in the manuscript.

Field-specific reporting

Please select the one below that is the best fit for your research. If you are not sure, read the appropriate sections before making your selection.

☒ Life sciences ☐ Behavioural & social sciences ☐ Ecological, evolutionary & environmental sciences

For a reference copy of the document with all sections, see nature.com/documents/nr-reporting-summary-flat.pdf

Life sciences study design

All studies must disclose on these points even when the disclosure is negative.

Sample size	no simple size calculation was performed. All available transcriptomic data related to vaccine response available on December 2020 was used in this manuscript.
Data exclusions	Transcriptomic data on children and juvenile (age < 18 years) was excluded from this analysis. Only human data was included. Those two exclusion criteria were pre-established.
Replication	10-fold cross-validation was used to assess the reproducibility of the classifier generated in the manuscript.
Randomization	No randomization was performed. All available samples were used to perform the meta-analysis. No potential confounders were identified using principal variance component analysis (PVCA).
Blinding	Blinding of the response data was not possible because this meta-analysis rely on publicly available data.

Reporting for specific materials, systems and methods

We require information from authors about some types of materials, experimental systems and methods used in many studies. Here, indicate whether each material, system or method listed is relevant to your study. If you are not sure if a list item applies to your research, read the appropriate section before selecting a response.

Materials & experimental systems

n/a	Involved in the study	
☒	☐	Antibodies
☒	☐	Eukaryotic cell lines
☒	☐	Palaeontology and archaeology
☒	☐	Animals and other organisms
☒	☐	Clinical data
☒	☐	Dual use research of concern

Methods

n/a	Involved in the study	
☒	☐	ChIP-seq
☒	☐	Flow cytometry
☒	☐	MRI-based neuroimaging
